# Delays during emergency obstetric care and their determinants among mothers who gave birth in South Gondar zone hospitals, Ethiopia. A cross-sectional study design

**DOI:** 10.1080/16549716.2021.1953242

**Published:** 2021-07-30

**Authors:** Gebrehiwot Ayalew Tiruneh, Mengstu Melkamu Asaye, Abayneh Aklilu Solomon, Dawit Tiruneh Arega

**Affiliations:** a Department of Midwifery, College of Medicine and Health Sciences, Debre Tabor University, Debre Tabor, Ethiopia; b Department of Women and Family Health, School of Midwifery, College of Health Science, University of Gondar, Gondar, Ethiopia

**Keywords:** Institutional delivery, delays, factor, obstetrics care, birth outcome

## Abstract

**Background:**

The majority of maternal deaths occur during delivery and the immediate postnatal period as a result of delays in seeking care, failure to reach health institutions, and receiving inappropriate health care. In developing countries, delayed access to timely healthcare contributes to high maternal mortality and morbidity.

**Objective:**

This study aimed to assess the delays during emergency obstetric care and associated factors with delays during emergency obstetric care.

**Method:**

A cross-sectional study design was conducted. We chose five hospitals at random in the South Gondar zone, Ethiopia. Face-to-face Interviews were conducted with 459 participants using a systematic sampling technique. For this analysis, bi-variable and multivariable logistic regression models were used. The Adjusted Odds Ratio was used to determine the statistical association with delays during emergency obstetric care at p-value <0.05 with a 95% confidence interval.

**Results:**

The proportion of delays during emergency obstetric care were found to be 59.7% in this study. The respondents’ mean age was 27.23 years old, with a standard error of 5.67. Pregnant mothers living in the rural areas (AOR: 4.1, 95%, CI: 2.36 to 6.25), no ANC visit (AOR: 1.8, 95% CI: 1.32 to 3.18), uneducated women (AOR: 4.6, 95% CI: 2.45 to 8.59) and referral to a higher level of care (AOR: 2.7, 95% CI: 1.60 to 4.44), were all significantly associated with delay.

**Conclusion:**

Delay during emergency obstetric care was found to be 59.7 percent. Rural residency, absence of ANC visit, uneducated mothers, and referred mothers from one level to the next level of care were factors that contributed to delays in emergency obstetric care in the study area.

## Background

Delays for utilizing emergency institutional delivery refer to the time from deciding to seek emergency care to the start of receiving health care service [1]. The first delay reflects the decision to seek care during pregnancy-related complications. It occurs at both the family and community levels. The second delay is the time it takes to reach a health care facility. The first and second delays are most common in rural areas. It happens where there is limited emergency transport, limited resources, and a reliance on public transportation. The third type of delay is the time it takes from arriving at the health care facility to receiving emergency labor and delivery treatment [2,3]. All three delays increase the risk of maternal morbidity and mortality. Each delay provides an opportunity to address the most appropriate conditions to ensure timely access to emergency maternity care [4].

Early detection and treatment of emergency conditions are protective factors during pregnancy, labor, and birth. They improve both the mother’s and the baby’s chances of survival [5,6]. Improved birth outcomes are associated with adequate supplies, medications, properly functioning equipment, skilled birth attendants, and a safe blood supply in the health facility [6]. Ethiopia has one of the highest maternal mortality rates in sub-Saharan Africa [7]. The delays caused by an inability to access emergent maternity care are associated with poor obstetric outcomes [8].

Every day, 830 women die as a result of pregnancy or childbirth complications around the world. Every year, more than 500,000 mothers die, with low-income countries accounting for 99% of maternal deaths [9,10]. According to the Ethiopian Demographic Health Survey (EDHS) report, the maternal mortality rate (MMR) in Ethiopia was 412 deaths per 100,000 live births [11]. The MMR is reduced by having access to skilled birth attendants and referral facilities for managing obstetric and neonatal complications. However, access to care is fragile particularly for those living in communities with impassable roads, limited transport options and resources [12,13].

Among several factors identified as barriers to pregnant mothers are limited access to health facilities and receiving early care from skilled birth attendants in a health facility. The perceived inequality of care received, an insufficient number of skilled health professionals, geographical inaccessibility, and disrespectful service delivery by health care providers are all factors associated with women not seeking health facility care. On the other hand, factors related to accessing skilled health care include a woman’s awareness of pregnancy danger signs that require emergent and competent health care management. Some families put pressure on women who lack autonomy in making health-care decisions by limiting access to timely emergency maternity care [2,3,14,15].

The international community is implementing a variety of strategies to reduce maternal mortality. Nonetheless, it remains a public health concern [16,17]. The new 2030 Sustainable Development Goals (SDGs) set a target of reducing global MMR to 70 per 100,000 live births [18]. Although tremendous progress has been made globally in reducing maternal deaths, some countries have made unequal progress. Notably, there has been a 47% decrease in maternal mortality worldwide from 1990 to 2010, but some countries, including Ethiopia, have reported slow progress [19].

The low utilization of modern health care services in Ethiopia is one explanation for poor health outcomes among mothers [17,20]. According to the EDHS, health institutions account for 48% of births [11]. Because of difficult-to-reach health-care settings, home births by unskilled birth attendants are common in rural areas. Almost half of all mothers are unable to access delivery services due to long travel times to a health facility, poor road construction, misconceptions about the value of health care services, and disrespectful behavior of health care providers. Nowadays, every pregnant woman is at risk of obstetric complications. Maternal death occurs most frequently between the third and sixth trimesters of pregnancy and 6 weeks after delivery. The condition becomes more complicated as access to skilled health care providers is delayed. Failure to seek quality care contributes to high maternal mortality rates during pregnancy and the postpartum period [9,14,21–26].

Both timely arrival and high-quality delivery services have been shown to reduce maternal and neonatal morbidity and mortality [14,27,28]. Exploring important variables for delays during emergency obstetric care in Ethiopia may thus be beneficial to policymakers and other interested partners. Furthermore, addressing maternal mortality and poor pregnancy outcomes helps in the identification of strategies that can improve access to skilled birth attendants, increase delivery services utilization, and promote health education about pregnancy danger signs for all women. Modifiable factors concerning such as early arrival at a health facility, emergent transportation, communication between both referring and receiving health teams, and ensuring competent, welcoming providers at all levels can improve outcomes and reduce mortality for mothers and babies. Listening to the unspeakable causes of delays and revising weak links and persistent gaps must be addressed collaboratively.

## Methods

### Study design and period

An institutional-based cross-sectional study design was used to assess delays during emergency obstetric care and associated factors among all mothers who gave birth in selected hospitals between June to July 2019.

### Study setting

This study was conducted in five selected hospitals in the South Gondar zone of Amhara Regional State, Ethiopia. Debre Tabor town serves as the zone’s capital. The town is located about 667 kilometers northwest of Addis Ababa and 103 kilometers northwest of Bahir Dar, the Amhara Regional State’s capital city. There are 2,609,823 inhabitants the administrative zone, including 1,304,911 female population [10]. One general government hospital (Debre Tabor General Hospital) and seven additional governmental hospitals (Mekane-Eyesus, Andabet, Nifas-Mewucha, Addis-Zemen, Tach Gait, Wogeda, and Event) serve the population, which is supplemented by 96 other public health centers. Agriculture is the main source of income for the majority of the population.

### Source population

All women who gave birth at South Gondar hospitals in 2019.

### Study population

All women who gave birth in randomly selected hospitals in the South Gondar zone during the data collection period.

### Eligibility criteria

- All women who gave birth in the South Gondar zone hospitals and lived for at least 6 months were eligible to participate in the study.
- Women who were pregnant or in the immediate postpartum period and refused to participate in the study had been excluded.
- Women who have chronic medical illness, unable to communicate, were triaged before being admitted to the hospital and waiting for the onset of labor were all excluded.

### Sample size determination

The sample size was determined using a single population proportion formula with the following assumptions: 76.3% [29] of the women who experienced delays to institutional delivery care, a 5% margin of error, 1.5 design effects, and a 10% non-response rate. We chose five hospitals at random in the South Gondar zone, Ethiopia. Interviews were conducted with 459 participants using a systematic sampling technique.

### Study variables

Dependent variable: Delays during emergency obstetric care

Independent variables:

- Socio-demographic characteristics: age, residence, marital status, ethnicity, religion, education of the mother, education of husband, occupation of the mother, occupation of husband, and family income.
- Obstetrics related factors: gravidity, parity, ANC follow-up, type of pregnancy, mode of delivery in the past, and the current mode of delivery.
- Health facility factors: available health facility, multiple referrals, the distance of health facility, means of transportation, road accessibility, previous pregnancy birthplace, know any danger signs of labor, and decision-maker for EOC [29,30],

### Operational definition

- **Delays during emergence obstetrics care**: refers to at least one or more delays from the three delays model [31]. Delays in seeking care: refers to the time it takes to seek care after the onset of labor that is longer than 1 hour [32].
- **Delays in reaching public health facility**: refer to a mother who is unable to arrive within 1 hour of walking to reach the health facility [33].
- **Delays in receiving appropriate care**: refers to a mother who does not receive an emergency obstetric care within the 5 min of arriving at a health facility.
- **Institutional delivery service**: when a mother gave birth in a health facility and was assisted by a skilled birth attendant, this is referred to as institutional delivery service.
- **Non-referred mothers**: a mother who gave birth among selected hospitals and had not been referred from another health care facility to the selected hospital for advanced care

### Data collection techniques

Face–to–face interviews were conducted with postpartum mothers in a private room before discharge. Women who were critically ill as a result of obstetric complications were not interviewed. Instead, their attendants were interviewed to include reports from those who have suffered as a result of delays in emergency obstetric care. In addition, the participant’s chart was reviewed to ensure that hospital records were cross-checked. Every day, the two-degree midwives supervised the five-diploma data collectors. We wrote the questionnaire in English and sent it to a linguist for revision. Then, for simplicity, we translated it to Amharic and then back to English to keep the consistency of the tool.

### Data quality assurance

One day of training was provided to data collectors and supervisors. The training was focused on the purpose of the study, data collection techniques, and data checks for completeness and consistency. The researchers were the only ones who could access the data, which was kept in a file cabinet. Pre-testing was performed on the questionnaire to ensure participant response, language clarity, and questionnaire appropriateness. At Wogera Hospital, data collectors interviewed 5% of the sample size to test the questionnaire. At the end of the test, ambiguous and culturally sensitive questions were amended, clarified, and adjusted before data collection began.

### Patient and public involvement

Meaningful participants were adopted this study in increase the relevance of the research work, enhance research excellence, and help ensure patient participation is as safe, sensitive, and ethical as possible. Their advice when designing, implementing, and evaluating research invariably makes studies more effective, more credible, and often more cost-efficient as well. It can provide health benefits to patients and their families by providing the satisfaction of having influenced care, being listened to, gaining additional insight into their issues, and providing social interaction and engagement.

### Statistical analysis

The data were coded and entered into Epi-data version 3.1. Then, we exported it to Statistical Package of Social Science (SPSS) version 20.00 for data checking, cleaning, and analysis. We used binary logistic regression to identify statistically significant independent variables. We used bi-variable and multivariable logistic regression models for this analysis. The independent variables having a p-value of less than 0.2 were entered into multivariable logistic regression for further analysis and to adjust confounding variables. The Adjusted Odds Ratio was used to determine the statistical association with delays during emergency obstetric care at p-value <0.05 with a 95% confidence interval.

## Results

### Sociodemographic characteristics

A total of 459 mothers voluntarily participated in this study among all mothers who gave birth the selected hospitals in the South Gondar zone. The mean age of the mothers was 27.23 years, with a standard deviation of ±5.67. Three-fourth (75.6%) of them were between the age groups of 20 and 34. In this study, 99.3% of the participants were married, 96.5% were Orthodox Christians, 24% worked for the government, and 37.5% lived in rural areas (Table 1). One hundred sixty-seven (36.6%) of study mothers were uneducated.

**Table 1.** Socio-demographic characteristics of participants in South Gondar Zone hospitals, Ethiopia, June

Variables	Categories	Frequency	Percentage (%)
Age (in years)	<20 20–34 ≥35	51 347 61	11.1 75.6 13.3
Residence	Urban Rural	287 172	62.5 37.5
Marital status	Married ^a^ Others	456 3	99.3 0.6
Ethnicity	Amhara ^b^ Others	457 2	99.6 0.4
Religion	Orthodox ^c^ Others	443 16	96.5 3.5
Education of the mother	Educated Uneducated	292 167	63.4 36.6
Occupation of the mother	Employed Unemployed	110 349	24.0 7.6
Education of the husband (N = 457)	Educated Uneducated	116 341	25.3 74.3
Occupation of the husband (N = 457)	Employed Unemployed	164 293	35.9 63.8
Family monthly income	≥2000 1001–1999 ≤1000	252 100 107	54.9 21.8 23.3

***NB****: ^a^ others: single and divorced, ^b^ others: Tigre and Oromo, ^c^ others: Muslim and protestant*

### Obstetric factors

Almost half (53.8%) of the study mothers were multi-para. About 85.5% of the study participants had antenatal follow-up visits. There were 30.3% referrals, 83.4% spontaneous vaginal delivery (SVD), and 4.6% stillbirth outcomes (Table 2). Of all of complications, 50% of mothers experienced severe vaginal bleeding complications after giving birth.

**Table 2.** Obstetrics related characteristics for delays during emergency obstetric care in South Gondar Zone hospitals, Ethiopia, June to July 2019

Variables	Categories	Frequency	Percentage (%)
Parity	Primi-para Multi-Para	212 247	46.2 53.8
ANC visits	Yes No	392 67	85.4 14.6
Number of ANC visit(n = 392)	Less than four Greater than four	253 139	64.6 35.4
Planned pregnancy	Yes No	302 157	65.8 34.2
Wanted pregnancy	Yes No	441 18	96.1 3.9
Time of labor start	Day Night	265 194	57.7 42.3
Ever had a history of home delivery (n = 247)	Yes No	125 122	50.6 49.4
Pregnancy outcome(n = 459)	Live birth Stillbirth	438 21	95.4 4.6
Arrived from which location	Other health facilities ANC ward Home/some else	139 9 311	30.3 2.0 67.8
The current mode of delivery	SVD Instrumental delivery C/S	383 30 46	83.4 6.5 10.0
Complication after delivery	Yes No	44 415	9.6 90.4
Type of complication after delivery (n = 44)	Severe vaginal bleeding High bleed pressure Fever Headache	22 12 4 6	50 27.3 9.1 13.6

### Health facility factors

In this study, 77.3% of participants used public transportation to get health care facilities (Table 3). About 17.75% of the transportation service was not available daily. Only 35.1% of laboring mothers used ambulance service (Table 4).

**Table 3.** Health-facility related factors for delays during emergency obstetric care in South Gondar Zone hospitals, Ethiopia, June to July 2019

Variables	Categories	frequency	Percentage (%)
Public transport service in your area to go to a health facility	Yes No	355 104	77.3 22.7
Frequency of transportation (n = 355)	Daily Not daily	292 63	82.25 17.75
Nearest health institution	Ye No	441 18	96.1 3.9
Referred from another health facility	Yes No	135 324	29.4 70.6
Mode of transportation	Ambulance Public bus ^a^ Other	161 100 198	35.1 21.8 64.4 3.1

*NB: ^a^ others: foot, local stretcher and Horse*

**Table 4.** Associated factors for delays during emergency obstetric care in South Gondar Zone hospitals, Ethiopia, June to July 2019

Variables	Delay to institutional delivery Yes No		COR (95% CI)	AOR (95% CI)
Residence Urban Rural	140 134	32 153	1.00 5(3.19–7.82)	1.00 4.1(2.40–7.01)*
Maternal education Educated Uneducated	145 129	147 38	1.00 3.(2.24– 5.28)	1.00 4.6(2.45–8.52)*
Maternal Occupation Employed Unemployed	219 55	130 55	1.00 1.6(1.1–2.59)	1.00 0.9(0.48–1.84)
Education of husband Educated Uneducated	90 184	26 157	1.00 1.8(2.95–4.80)	1.00 0.8(0.40–1.49)
Occupation of husband Employed Unemployed	82 192	82 101	1.00 1.9(1.28– 2.80)	1.00 0.9(0.51–1.47)
ANC visit Yes No	220 54	172 13	1.00 2.3(1.54– 3.40)	1.00 1.8(1.32–3.18)*
Number of ANC visit Greater than four Less than four	86 134	53 119	1.00 3.3(1.61–6.63)	1.00 2.1(0.05–5.17)
Planned of pregnancy Yes No	201 101	73 84	1.00 3.2(1.71– 6.14)	1.00 1.3(0.59–2.95)
Time of labor start Day Night	182 83	83 102	1.00 2.4(1.66–3.57)	1.00 1.8(0.17–2.85)
Referred from another health facility to the selected hospital Yes No	126 148	31 154	4.3(2.69– 6.65) 1.00	2.7(1.59–4.44) * 1.00

***NB:*** *indicates p-values ≤ 0.001

### Delay during emergency obstetric care and associated factors

The delay during emergency obstetric care is 59.7% (95%, CI: 54.80 to 64.30). In the bi-variable analysis, rural residence, educational status of mothers, husband’s education status, occupational status of mothers, husband’s occupational status, women who had no ANC visit, number of ANC visits, planned pregnancy, and time of onset of labor and referral cases (women who were referred from another health care facility for advanced care) statistically associated variables with delays during emergency obstetric care at P-value =0.2.

The associated variables entered into a multivariable analysis involving a logistic regression model to control confounding variables (Table 4). Only rural residency, no ANC visit, uneducated mother, and referral cases were predictive variables for delays in emergency obstetric care in multivariable analysis (P-value <0.05). The odds of rural residence for the risk of delays during emergency obstetric care was 4.1 (AOR = 4.10, 95% CI: 2.36 to 6.25) higher compared to their counterparts. The odds of the absence of ANC visit for the risk of delays during emergency obstetric care was 1.8 (AOR = 1.8, 95% CI: 1.32 to 3.18) higher compared to those who had at least one ANC follow-up. The odds of non-educated mothers being at risk of delays during emergency obstetric care was 4.6 (AOR = 4.6, 95% CI: 2.45 to 8.59) higher compared to their counterparts. The odds of the presence of referral cases from other health institutions for the risk of delays during emergency obstetric care was 2.7 (AOR = 2.70, 95% CI: 1.60 to 4.44) higher compared to non-referred mothers.

## Discussion

The study aimed to assess the proportion of delays during emergency obstetric care among all mothers who gave birth in South Gondar zone hospitals. The current study revealed that 59.7% of mothers had delays during emergency obstetric care. The proportion of to emergency obstetric care was high as compared to the first (46.80%), second (44.00%), and third (31.70%) maternal delays in Gamo Zone, Southern Ethiopia [34]. On the other hand, the delay during emergency obstetric care was lower than in other studies conducted in Yem-special Woreda [29], Pakistan [32], and Mozambique [33] which were 76.3%, 70%, and 69.7%, respectively. It could be related to differences in study participants’ socioeconomic and cultural position, as well as different study approaches. However, it was consistent with a finding in Myanmar (55.6%) [30], but higher than another study conducted in Brazil (53.8%) [35]. In this study, 63.4% of the women and 74.3% of her husband were uneducated. The possible reason might be the difference in the educational status of the mothers and their husbands in Ethiopia and Brazil. The current study found that 37.5% of participants were from rural areas and had a poor socioeconomic status. It could have an impact on how they use delivery services and contribute to a high proportion. In this study, only rural residency, no ANC visits, uneducated mothers, and referral cases were predictive variables for delays during emergency obstetric care in multivariable analysis.

Mothers in rural areas are 4.1 times more likely to be delayed during emergency obstetric care than urban residence. It was consistent with a study conducted in the Gurage zone[32] and the Oromia region in Ethiopia [36]. In addition, 37.5% of participant in the current study lived in rural areas and had low socioeconomic status. It may have an effect on their delivery service utilization and contribute to the high proportion. It may be due to a lack of skill among health care providers and a disrespectful service delivery system [34]. The other possible reasons may be lack of women empowerment for early decision-making autonomy, poor physical access to health facilities that provide safe delivery service, poor road construction, and lack of access to health education regarding complications during labor and delivery [14]. Cultural and restrictive socio-cultural norms influence rural mothers that may hurt the utilization of maternal health care services. Urban mothers are aware of the benefits of health facility delivery service through different media. So, they are less prone to delays of institutional delivery [37].

The result of this study revealed that mothers who had no ANC visits were 1.8 times more likely to be delayed by emergency obstetric care as compared to those who had at least one ANC visit. This finding is in agreement with a study conducted in Kaffa and Sheka, Ethiopia [38–40]. One possible reason might be a lack of awareness of the danger signs and disrespectful service delivery system of health care professionals.

The study also found that uneducated mothers were 4.6 times more likely to be delayed in emergency care as compared to educated mothers. This finding was consistent with previous research in the Hadiya Zone [14], and Yem special Woreda [29]. The possible explanation may be the accessibility of services to information about the advantages of institutional delivery, pregnancy-related complications, knowledge of birth preparedness, and awareness of danger signs. Non-educated women are also less likely to have antenatal visits and did not empower themselves in making decisions to seek care when they faced emergencies during delivery. They are also more likely to be influenced by traditional beliefs and cultures and exposed to labor and delivery-related complications secondary to delays to institutional delivery services [37].

Mothers referred from other health care facilities were three times more likely to be delayed by emergency obstetric care than non-referred mothers (a woman who was not referred from other health care facility and gave birth among the selected hospital). The findings are comparable to a study undertaken in Ethiopia’s Hadiya Zone [14] and Mozambique [33]. A plausible reason is that each has a similar study design and socio-demographic features of study participants. In general, inadequate financial and logistical support, a severe shortage of skilled human resources for health, high staff attrition, and an ineffective referral mechanism [38], have all harmed physical access to health services, potentially leading to inappropriate health-seeking behavior [37]. As a result, improving access to health care facilities and addressing the associated factors at the community level through synergistic intervention will reduce the proportion of delays during emergency obstetric care.

### Strengths and limitations of this study

- We employed the probability sampling technique to ensure that the findings could be generalized to the study population.
- We conducted analyses using a logistic regression model, demonstrating the relationship between predicted and response variables.
- The model controlled the effect of confounders to prevent bias from being introduced at the analysis stage
- However, postpartum mothers who were eager to leave the hospital may have caused information bias.

## Conclusion

Delay during emergency obstetric care was found to be 59.7%. It was high compared to other similar studies. Rural residency, absence of ANC visit, uneducated mothers, and referred mothers from one level to the next level of care were factors that contributed to delays in emergency obstetric care in the study area.

## Paper context

A total of 459 postpartum women volunteered to be a part of this study. Although much has been done to reduce maternal mortality and disability related pregnancy and delivery, health systems in low-income countries continue to face challenges. As a result, our research has acted as a lens for stakeholders to rethink new tactics for reducing morbidity and mortality during the first stage of emergency obstetrics care.
